# UA_L-DoTT: University of Alabama’s large dataset of trains and trucks

**DOI:** 10.1016/j.dib.2022.108073

**Published:** 2022-03-22

**Authors:** Maxwell Eastepp, Lauren Faris, Kenneth Ricks

**Affiliations:** The University of Alabama, Tuscaloosa, AL 35487, United States

**Keywords:** LiDAR, Camera, Trains, Trucks, Deep Learning

## Abstract

UA_L-DoTT (University of Alabama’s Large Dataset of Trains and Trucks) is a collection of camera images and 3D LiDAR point cloud scans from five different data sites. Four of the data sites targeted trains on railways and the last targeted trucks on a four-lane highway. Low light conditions were present at one of the data sites showcasing unique differences between individual sensor data. The final data site utilized a mobile platform which created a large variety of viewpoints in images and point clouds. The dataset consists of 93,397 raw images, 11,415 corresponding labeled text files, 354,334 raw point clouds, 77,860 corresponding labeled point clouds, and 33 timestamp files. These timestamps correlate images to point cloud scans via POSIX time. The data was collected with a sensor suite consisting of five different LiDAR sensors and a camera. This provides various viewpoints and features of the same targets due to the variance in operational characteristics of the sensors. The inclusion of both raw and labeled data allows users to get started immediately with the labeled subset, or label additional raw data as needed. This large dataset is beneficial to any researcher interested in machine learning using cameras, LiDARs, or both. The current dataset is publicly available at UA_L-DoTT.

## Specifications Table

**Table:** 

Subject	Artificial Intelligence
Specific subject area	Deep Learning; Remote Sensing; Large Vehicle Detection; Semantic Segmentation
Type of data	LiDAR based point clouds
	Camera images
	Train car and truck location annotations
	POSIX timestamps
How data were acquired	Data was acquired using a custom sensor mount designed to support all LiDARs and the camera simultaneously. The sensors and software used for data collection include
	LiDARs:
	Velodyne VLP-16 Puck
	Velodyne VLP-16 Hi-Res
	Velodyne Puck 32MR
	Ouster OS1-16 Gen1
	Ouster OS2-64
	Camera:
	FLIR Blackfly S GigE BFS-PGE-31S4C-C
	Software:
	Custom LiDAR Driver
	Spinnaker SDK
Data format	Raw LiDAR data: *PCD*
	Raw images: *JPG*
	LiDAR annotations: *PCD*
	Image annotations: *TXT*
	Timestamps: *TXT*
Parameters for data collection	Data was collected with stationary and mobile sensors relative to the targets of interest. Velodyne sensors operated at a sampling rate of 8 Hz resulting in a horizontal angular resolution of 0.159${}^{\circ }$. Ouster sensors operated at 10 Hz, resulting in a horizontal angular resolution of 0.176${}^{\circ }$. The Blackfly camera also operated at 10 Hz. Collections typically occurred mid-day, but one collection occurred during low-lighting conditions. Any changes to these parameters are outlined in the individual site sections.
Description of data collection	The LiDAR data was collected from the above sensors utilizing a custom driver executing on a laptop running Linux 20.04. This driver collected and saved LiDAR scans simultaneously allowing the individual sensors to be loosely time synchronized. All sensors were mounted to a custom mounting frame attached to a tripod or truck. The mount was designed to reduce interference between the various sensors. Camera data was collected utilizing the Spinnaker SDK provided by the camera manufacturer. The camera was connected to a separate laptop running Windows 10, and was mounted to the same sensor mounting frame.
Data source location	Institution: The University of Alabama
	City/Town/Region: Tuscaloosa, AL
	Country: United States
Data accessibility	Repository name: Figshare+ Data identification number: 10.25452/figshare.plus.19311938.v1[1] Direct URL to data: https://doi.org/10.25452/figshare.plus.19311938.v1

## Value of the Data

• This dataset is useful to those in the machine learning community, the transportation industry, autonomous vehicle researchers, and those using cameras, LiDARs, or both for object classification.
• Those working in the railway industry will find this data useful for the development of tracking and monitoring systems, such as those in [2].
• Researchers developing algorithms to fuse camera and LiDAR data will find this dataset beneficial in testing and validating their system, such as those in [3].
• This data can be utilized to develop machine learning algorithms intended to be trained with LiDAR data such as those in [4]. It can also be used for training machine learning algorithms to identify trains, trucks (already labeled), or other objects (labeled by users).
• This dataset was created with a diverse suite of sensors. All sensors collected loosely synchronized data of the same targets. Time synchronized data allows for comparison of the different data collection systems. This could further support the research done in [5].

## 1 Data Description

### 1.1 File organization

**Fig. 1:**
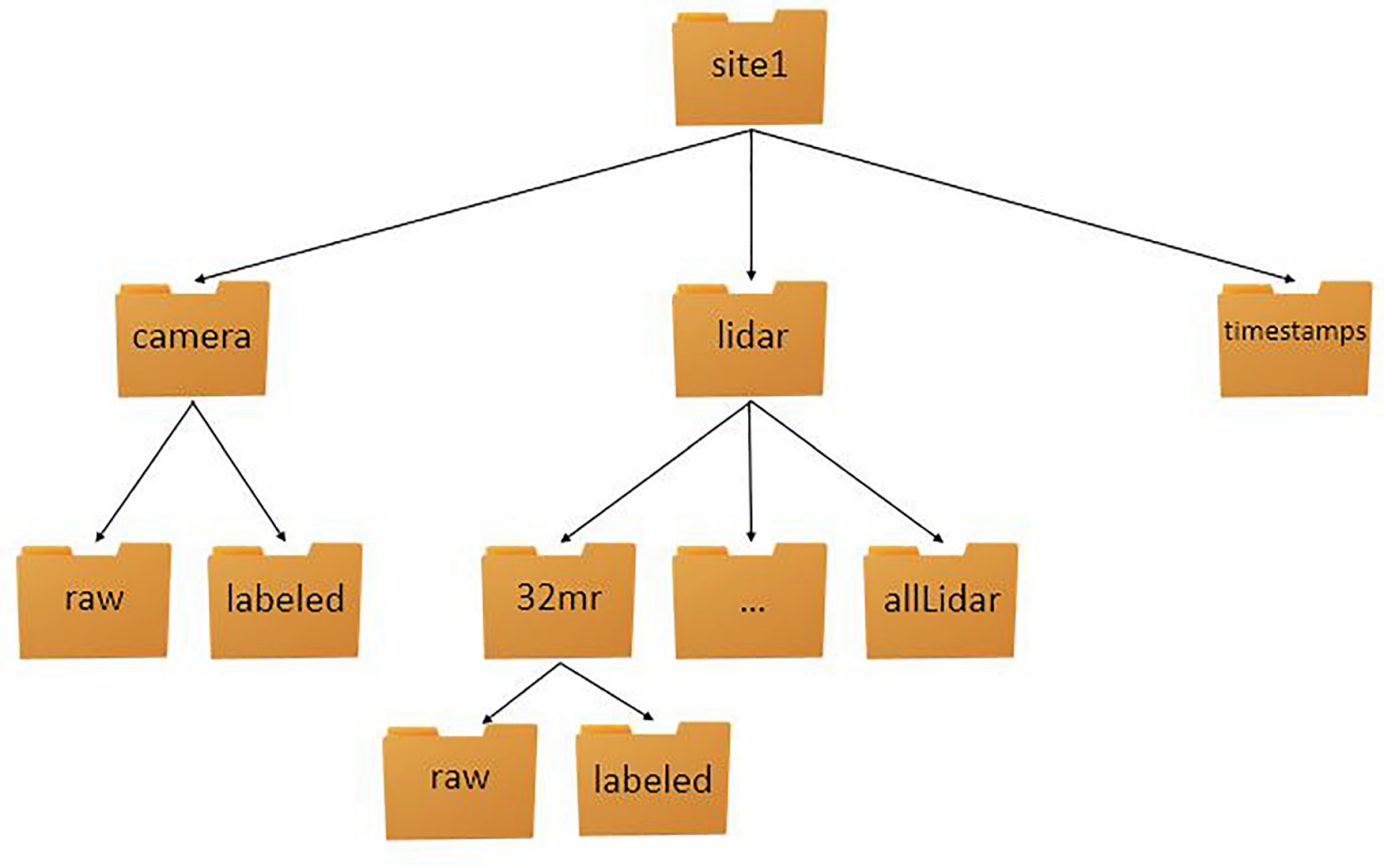
UA_L-DoTT Dataset Folder Hierarchy.

Figure 1 outlines the Site 1 folder organization of the UA_L-DoTT dataset. The entire dataset is divided into the five different sites and each site folder is organized similarly. For each site, the data is separated into “camera”, “lidar”, and “timestamps” sub-folders. The “camera” folder contains sub-folders for “raw” and “labeled” data. The “lidar” directory contains separate folders for each sensor as well as an “allLidar” folder, which contains the raw and labeled scans for every LiDAR at that site. The “timestamps” folder contains all the timestamp information at the site.

### 1.2 Naming convention

Table 1 outlines the naming convention that the camera and LiDAR data utilize. Each file descriptor is separated by an underscore. Note that for all truck related scans there are no associated train numbers. This is further discussed in the Site 3 subsection of Experimental Design Materials and Methods. Additionally, scan numbers are sequential relative to their POSIX timestamp.

**Table 1:** File naming convention.

File Descriptor	Purpose	Examples
Sensor name	Sensor used	*32mr, puck, blackfly, etc.*
Target	Object the scan is targeting	*train, truck*
Train number	Scanned train	*000, 001, 002, etc.*
Scan number	Scan number from the specified train	*00000, 00001, 00278, etc.*
Scan type	Raw or labeled scan	*raw, labeled*

Example: *32mr_train_013_00173_labeled.pcd*

Timestamp files utilize their own naming convention with only two fields: site and sensor. Each are separated by underscores as well. There are timestamp files associated with each of the individual sensors that were utilized at a given site. For example, the Site 2 timestamps for the 32MR are, “site2_32mr_timestamps.txt”. Additionally, there is a file that contains all the timestamps from all the LiDARs at that site.

### 1.3 LiDAR data

The LiDAR scans utilize the Point Cloud Data (PCD) file format, which is a standard file format natively supported by the Point Cloud Library (PCL) [6] and MATLAB. Additionally, it can be opened by most text editors. All scans utilize the ASCII standard within the PCD format to facilitate ease of use within the Python language (commonly utilized for machine learning applications) and to make them more human readable. This dataset includes two types of LiDAR scans: raw and labeled. These can be distinguished by both the file name and the contents of the file. There is a header for each type that complies with the standard PCD format. Raw scans are formatted as XYZI and labeled scans are formatted as XYZLO for each data point. Individual data points are separated by a newline. A description of these fields is outlined in Table 2. Note that the ‘object’ field is a legacy field from the labeling process, and will be -1 for every point.

**Table 2:** Point cloud field descriptions.

Field	Utilized By	Data Type	Description	Example
X	Raw, labeled	Float	Spatial coordinate	*3.4962714*
Y	Raw, labeled	Float	Spatial coordinate	*12.380303*
Z	Raw, labeled	Float	Spatial coordinate	*-0.039474506*
I (Intensity)	Raw	Float	Light intensity	*36*
L (Label)	Labeled	Integer	Classification	*0, 1*
O (Object)	Labeled	Integer	Legacy field	*-1*

**Fig. 2:**
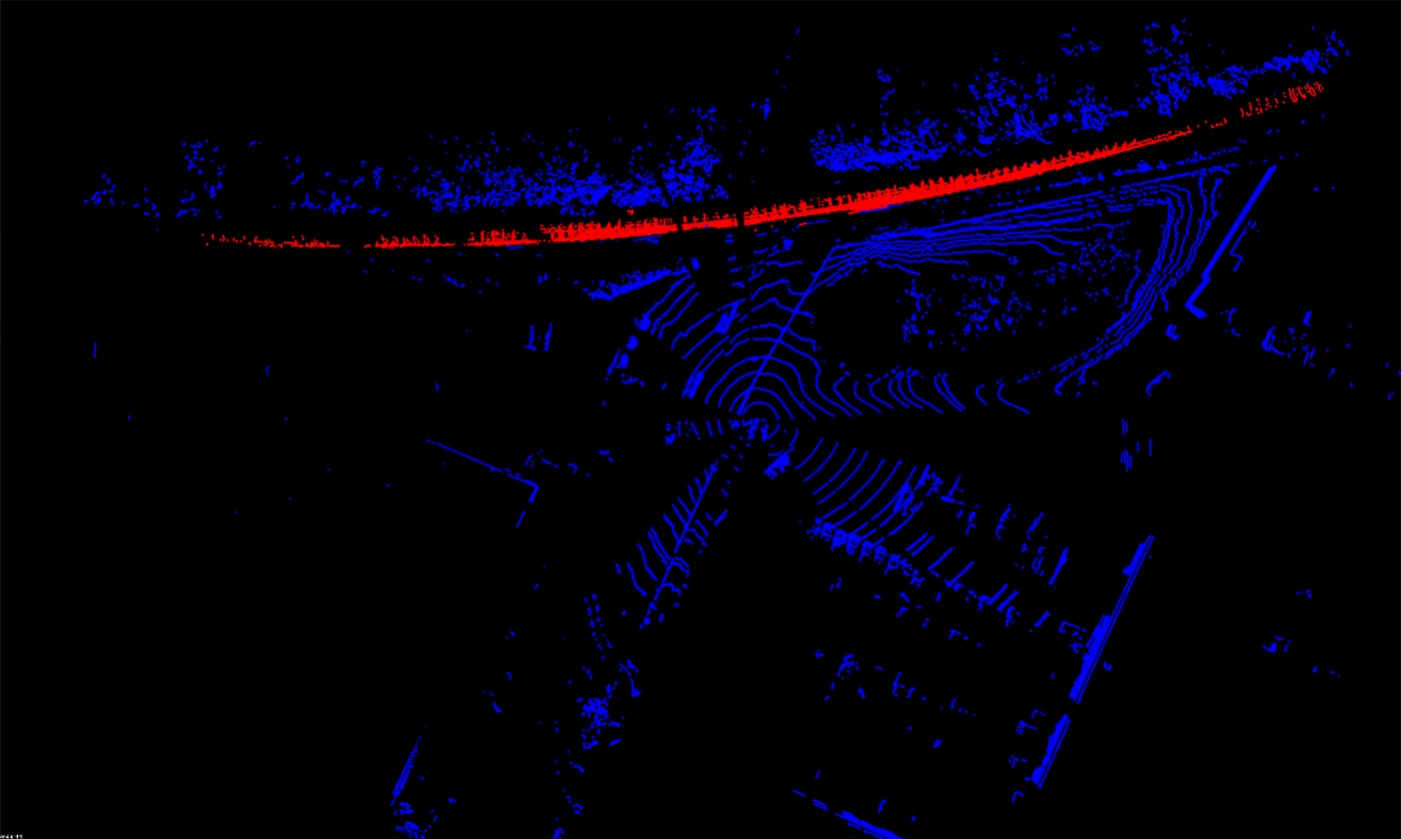
Example of Labeled LiDAR Scan at Site 2.

Figure 2 shows an example of a labeled point cloud displayed using a visualization tool. The points belonging to a labeled target are shown in red to easily identify them. The labeled files do not include LiDAR intensity data. However, the naming convention enables the recovery of the intensity data by correlating the raw and labeled files of the same name.

### 1.4 Camera data

**Fig. 3:**
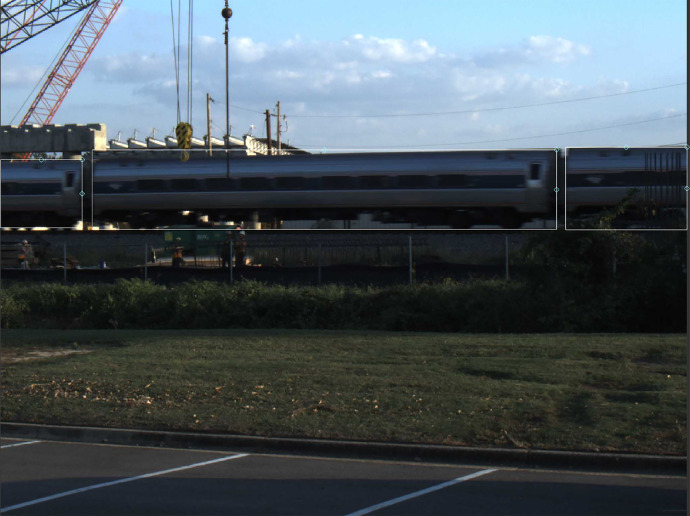
Example of Labeled Image from Site 4.

Raw camera images utilize the JPEG image standard. The corresponding labeled image data is saved in text files. These text files consist of information describing the rectangular, axis-aligned bounding boxes drawn around targets of interest in the raw images. An example of these bounding boxes overlaid with an image can be seen in Fig. 3. White boxes surround the full train car in the center, and the two partial train cars on either end. The text files contain four integers separated by commas in the format of XYWH for the bounding box that is created for each target. XY represents the coordinates of the bounding box’s top left corner and WH represents the width and height of the bounding box in pixels. The (0, 0) coordinate is assumed to be the top left corner of the image.

**Fig. 4:**
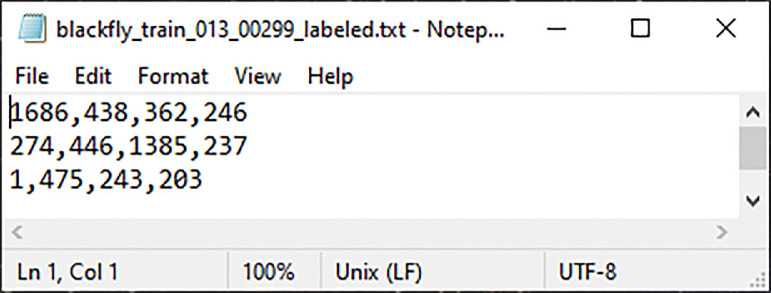
Example of an Image Annotations File from Site 4.

When multiple targets exist within an image, each bounding box within the text file is separated by a newline. If the image is a negative scan, then an empty text file containing no targets is created. The contents of one such label file from Site 4 can be seen in Fig. 4, which also showcases an example of the naming convention utilized for camera labels. This label file corresponds to the image shown in Fig. 3.

### 1.5 Timestamps

**Fig. 5:**
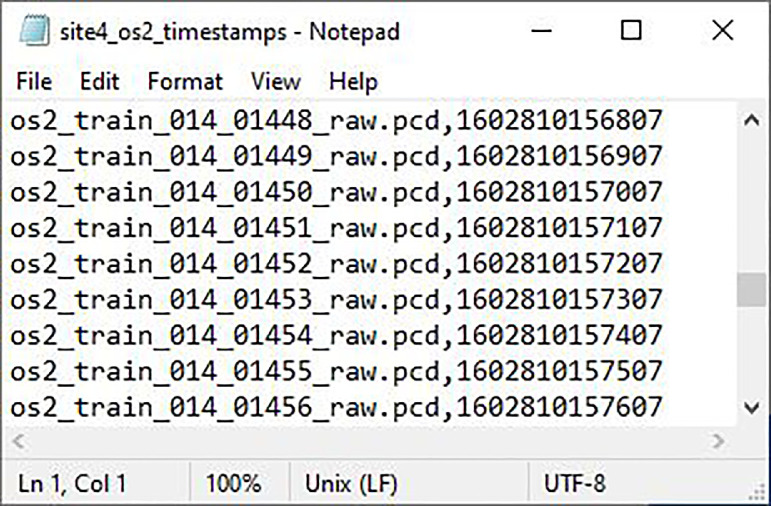
Subsection of the Site 4 OS2 Timestamps File.

Timestamp files contain a list of the raw file names for the associated sensor with each file’s corresponding POSIX timestamp. The timestamp files allow users to correlate different sensor scans and camera images to help visualize the point clouds. A subsection of the OS2 timestamp file for Site 4 can be seen in Fig. 5, also showcasing an example of the naming convention utilized for timestamp files.

## 2 Experimental Design, Materials and Methods

This dataset was collected across five different sites. Each one had a slightly different collection methodology that matured from site to site. These differences are discussed in the individual site sections below. More information regarding site locations and universal sensor parameters is discussed in Collection Parameters.

For train sites, collection began when the train horn was heard, and stopped when the train was no longer in sight. This provided negative scans (scans where no target of interest exists within it) before and after the train that were integrated into the dataset. For the truck site, data was collected continuously as trucks passed through the sensor field-of-view (FOV). As such, there are many more negative scans. This required careful selection of data to be labeled to create a more meaningful dataset, discussed further in the Site 3 section.

For collections, all sensors were mounted to a custom built frame, which was then mounted to tripods or a mobile platform. This mount was built using t-slot aluminum extrusion which was anodized black. This held and elevated the sensors in a stationary position throughout the collection. Sensors were re-arranged from site to site, so details of positioning are discussed in the individual site sections. Upright members assisted in preventing interference between the various sensors, with the black anodizing helping to further mitigate this. These uprights also allowed the sensors to be positioned to create vertical scan lines. Any instances of this are discussed in the individual site sections.

### 2.1 LiDAR collection

During collections, all LiDARs were connected to a 1-gigabit network switch. This was then connected to a laptop so all LiDARs were communicated with simultaneously. A custom driver was written in C++ that could collect a scan from each sensor and save it directly to the disk in PCD format. This driver evolved with each collection as issues were identified and corrected. These changes and how they impacted the overall collection are outlined in each of the site sections.

The driver saved each scan with a POSIX timestamp in the filename. After collection, the scans were renamed to meet the previously discussed naming convention, and the timestamps were used to create the timestamp files mentioned previously.

Any saved LiDAR scan that did not include at least 2,000 points (representing approximately 90% of the horizontal points) was categorized as an incomplete scan and was discarded. Therefore, all point clouds in this dataset have a width of at least 2,000 points. A summary of the percentage of incomplete scans across all LiDARs at each site is outlined in Table 3. Detailed explanations for the amount of incomplete scans are discussed in each individual site section. Incomplete scans can result from errors saving the file or from problems with the LiDAR collecting the scan.

**Table 3:** Percent of incomplete LiDAR scans.

Site	Incomplete LiDAR Scans
Site 1	67.23%
Site 2	3.26%
Site 3	12.94%
Site 4	1.44%
Site 5	0.37%

To label the point clouds, a custom labeling tool was written in C++ utilizing PCL to annotate them. This tool allowed the research team to go through and manually select any points that were associated with a train or truck. The tool then auto-generated the PCD label file with the previously discussed fields.

### 2.2 Camera collection

The Blackfly camera was equipped with a lens with a focal length of 16 mm and an optical format of 1/1.8”. At each site, the camera was connected to the same 1-gigabit network switch as the LiDARs. It was then connected to a laptop executing Windows 10 for interfacing. SpinView 2.0.0.146 included in the Spinnaker SDK provided by the manufacturer was used on the laptop to capture images. Within the software, the acquisition frame rate was enabled and set to 10 FPS to match the speed of the Ouster LiDARs. The recording mode was set to buffered to minimize dropped frames. The compression quality on a range from 0–100 was set to the default of 75. This prioritized image quality over image compression while still reducing overall file size. Additional settings were adjusted for low lighting conditions, discussed in the Site 4 section.

Labeled data was produced with a custom labeling tool. This tool enabled manual labeling by clicking on two opposite corners of the target of interest. This created the axis-aligned bounding box. Users adjusted the box size and position to create a more accurate label. The tool then auto-generated the text files based upon the labels.

### 2.3 Collection parameters

Table 4 outlines details for each data collection. It covers the date, time, latitude, and longitude of each data site. The height of the sensors at each site was additionally recorded to provide a precise sensor location in relation to the ground.

**Table 4:** Collection parameters.

Site #	Date & Time	Latitude & Longitude	Sensor Height (m)
1	06/02/2020 10:30am–3:30pm	(33.202769, -87.528605)	1.330
2	07/30/2020 10:30am–2:30pm	(33.200668, -87.553916)	1.380
3	09/11/2020 10:30am–2:00pm	(33.182356, -87.614442)	1.380 & 1.730
4	10/15/2020 3:30pm–9:30pm	(33.202183, -87.538140)	1.870 & 2.210
5	11/16/2020 11:00am–3:00pm	(33.202183, -87.538140)	1.090 & 1.470

The distance from the sensors to the targets across all sites ranged from 42 m–240 m. Site specific distance ranges are noted in each site section. All data collection was done during sunny to partly cloudy weather except for nighttime collection at Site 4. Minor occlusions existed within the FOV of every site including foliage, utility poles, road signage, chain-link fences, construction equipment, and automobiles.

Table 5 outlines relevant LiDAR specifications. Namely, the number of scan lines of the individual LiDARs, the maximum range, and the rotational rate of each LiDAR are recorded. The LiDARs utilized at each site are also outlined here. The Ouster LiDARs and the camera had a fixed horizontal resolution of 2,048 at 10 Hz. The specific rotational rate of the Velodyne LiDARs was then configured to match the other sensors as closely as possible, resulting in a collection frequency of 8 Hz.

**Table 5:** LiDAR parameters.

LiDAR	Sites Used	Scan Lines	Range (m)	Rate (Hz)
Puck	1–5	16	100	8
Hi-Res	1–5	16	100	8
32MR	1–5	32	120	8
OS1	1–5	16	120	10
OS2	3–5	64	240	10

At each site, all sensors collected data using their base collection rates shown in Table 5. At some sites down sampling was used to manage the number of scans collected or labeled. This down sampling lowered the rates of collected or labeled data from these sites. However, due to the slow speeds of the targets, the resulting data is still loosely synchronized and correlated to allow for cross-sensor comparisons of the same targets.

### 2.4 Site 1

**Fig. 6:**
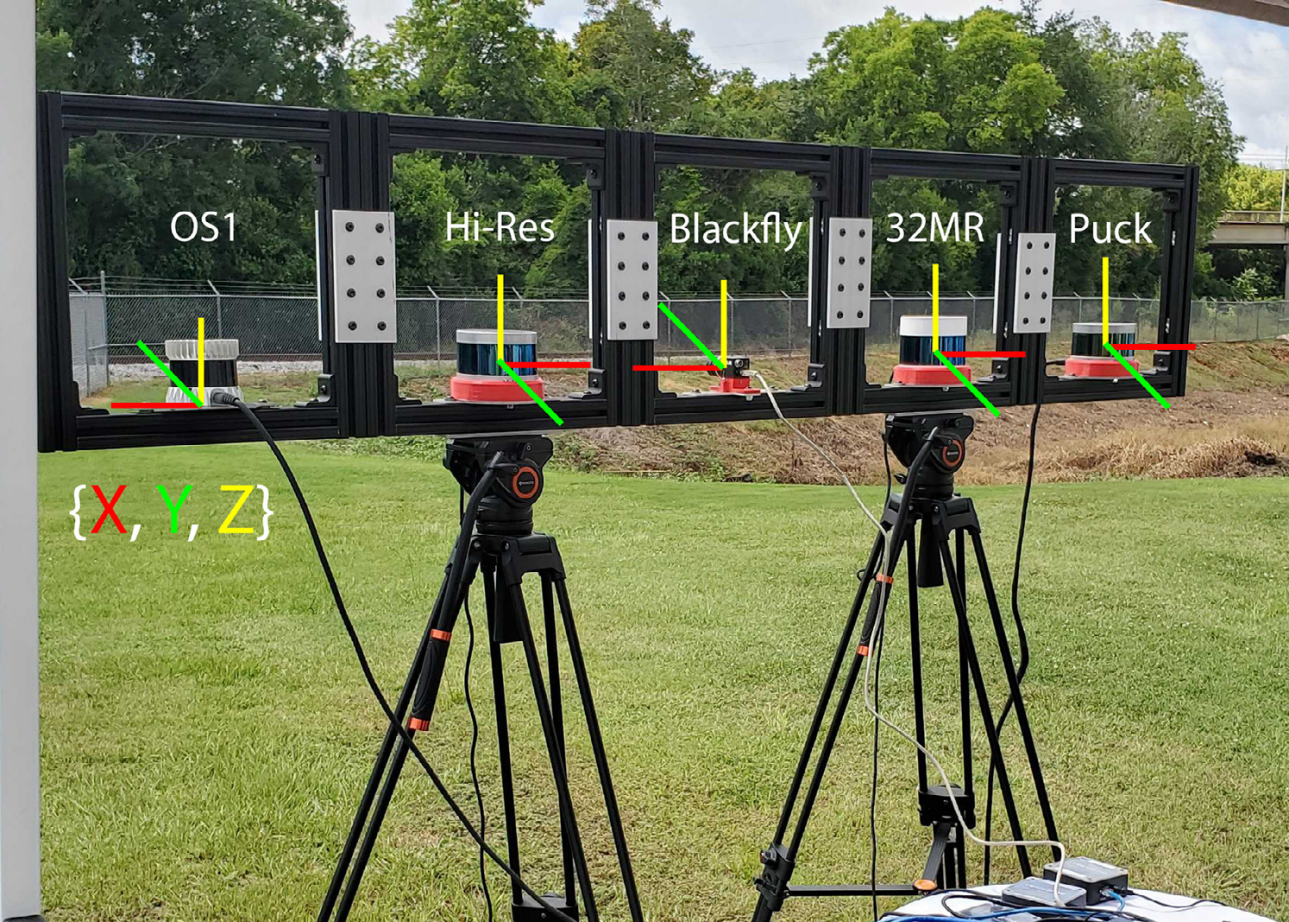
Sensor Arrangement for the First and Second Sites.

Site one targeted six trains running on two separate parallel tracks. Distance to targets of interest ranged from 42m–93m. The sensors were stationary as the trains moved both left-to-right and right-to-left through their FOV. The height of the sensors is specified in Table 4. The location of the sensors on the sensor mount were similar to those at Site 2 (seen in Fig. 6). The position of each sensor relative to the camera’s coordinate frame are summarized in Table 6. Additional coordinate system details are described in the Site 2 section. A summary of the total scans collected and those that were labeled can be seen in Table 7.

**Table 6:** Site 1 relative coordinates.

Sensor	Relative Coordinate (m)
Blackfly	(0, 0, 0)
Puck	(-0.610, -0.015, 0.018)
Hi-Res	(0.305, -0.015, 0.018)
32MR	(-0.305, -0.015, 0.017)
OS1	(0.610, -0.015, -0.032)

**Table 7:** Site 1 data summary.

Sensor	Raw Scans	Labeled Scans
Blackfly	6,027	1,565
Puck	359	359
Hi-Res	4,849	4,849
32MR	533	533
OS1	2,562	2,562

### 2.5 LiDAR details

During this collection, it was found that a significant portion of the scans were incomplete, as outlined in Table 3. It was determined that this was due to bandwidth limitations when trying to save scans from four simultaneous LiDARs. For an undetermined reason, the driver favored the Hi-Res and OS1. As such, there are minimal scans from the 32MR and Puck at this site. Fixes were implemented to mitigate this in future collections.

As mentioned, the custom mount allowed for LiDARs to be attached vertically, creating vertical scan lines. This was the only site where this was done. While changing orientations, trains came through that resulted in a mix of horizontal, vertical, or missing sensors during the changes. Due to the unique combination of different LiDAR positions varying between trains at this site, the orientation for each sensor is outlined in Table 8.

**Table 8:** Sensor orientations for site 1.

Train	Vertical Sensors	Horizontal Sensors	Missing Sensors
0	None	All	None
1	None	All	None
2	32MR	Blackfly, Puck, Hi-Res, OS1	None
3	32MR	Blackfly	Puck, Hi-Res, OS1
4	Puck, Hi-Res, 32MR	Blackfly	OS1
5	All LiDARs	Blackfly	None

When labeling the data, the static relationship between the trains and the LiDARs was exploited. Since trains will always appear in the same region, a script was written to automatically label every point within a volume aligned with the train tracks. This script was executed on every point cloud collected at this site. Afterwards, an annotator checked the point clouds and corrected any incorrectly identified outliers. However, this system did not work for the scans taken with vertical sensors. As a result, every vertical scan was manually labeled.

### 2.6 Camera details

During the first data collection, the Blackfly was manually zoomed and cropped to a resolution of 2,048 × 600 so that trains would occupy the majority of the FOV. Since the camera remained static between trains, this was only done once, and remained the same for all trains at this site. This zoom and crop operation was only performed at Site 1, as it was realized that this resolution may not be consistent with future sites. Additionally, the zoom and crop operations limit the usefulness of the images for machine learning applications and other potential use cases. As such, the Blackfly was reverted to its full resolution of 2,048 × 1,536 for all remaining data sites, and no form of zooming or cropping was utilized. Negative images were not included in this site’s dataset. Negative images were included at all subsequent data sites.

When initially labeling the camera data for Site 1, every image was manually labeled. It was realized that due to the relatively slow speed of the trains and the much higher sampling rate of the camera, many images could be considered “pseudo-duplicates”. The train(s) moved so slowly between frames that the images were nearly identical to one another. Thus, annotators only labeled every 3-7 images. It was at the discretion of the labeler to determine when the scene had changed drastically enough to warrant a new label.

### 2.7 Site 2

This site targeted seven trains using three separate parallel railroad tracks. Distance to targets of interest ranged from 48m–76m. The sensor arrangement was the same as that from Site 1, with the height of the sensors being outlined in Table 4. The overall setup along with annotated coordinate systems for the sensors can be seen in Fig. 6. For further clarification, the X-axis is aligned with the lower support member of the mount (parallel); the Y-axis is directed away from the mounting system (perpendicular); and the Z-axis is always directed towards the sky. Details of each sensor’s position relative to the camera’s coordinate frame are outlined in Table 9. A summary of the scans collected at Site 2 and those that were labeled are outlined in Table 10.

**Table 9:** Site 2 relative coordinates.

Sensor	Relative Coordinate (m)
Blackfly	(0, 0, 0)
Puck	(-0.762, -0.015, 0.018)
Hi-Res	(0.381, -0.015, 0.018)
32MR	(-0.381, -0.015, 0.017)
OS1	(0.762, -0.015, -0.032)

**Table 10:** Site 2 data summary.

Sensor	Raw Scans	Labeled Scans
Blackfly	14,185	4,675
Puck	4,574	4,574
Hi-Res	4,144	4,144
32MR	3,599	3,599
OS1	5,175	5,175

### 2.8 LiDAR details

At this collection, the driver was modified to only save every third scan. This effectively reduced the collection speed of the LiDARs down to 2.67 Hz and 3.33 Hz for the Velodyne and Ouster LiDARs respectively. However, this mitigated the bottleneck associated with saving the scans, resulting in significantly more valid scans, as seen in Table 3. The 32MR had the largest amount of incomplete scans, which can be attributed to the bandwidth issue not being completely resolved. The remaining incomplete scans can be attributed to inconsistencies in sensor operation.

While labeling, the static relationship between targets and sensors was again exploited to automatically label every scan taken. As with Site 1, these scans were then manually verified and corrected if need be.

### 2.9 Camera details

There were no changes with the camera data collection at this site. Thus, the camera base frame rate of 10 Hz was used. Due to the long stretch of train tracks, the camera was occasionally panned to the left and right to display an extended view of the tracks. This added variety to the data collected from this site. When labeling the images for Site 2, the same final methodology used at Site 1 was adopted. It was at the discretion of the annotator to label every 3-7 images based on their determination of when the scene had changed significantly.

### 2.10 Site 3

**Fig. 7:**
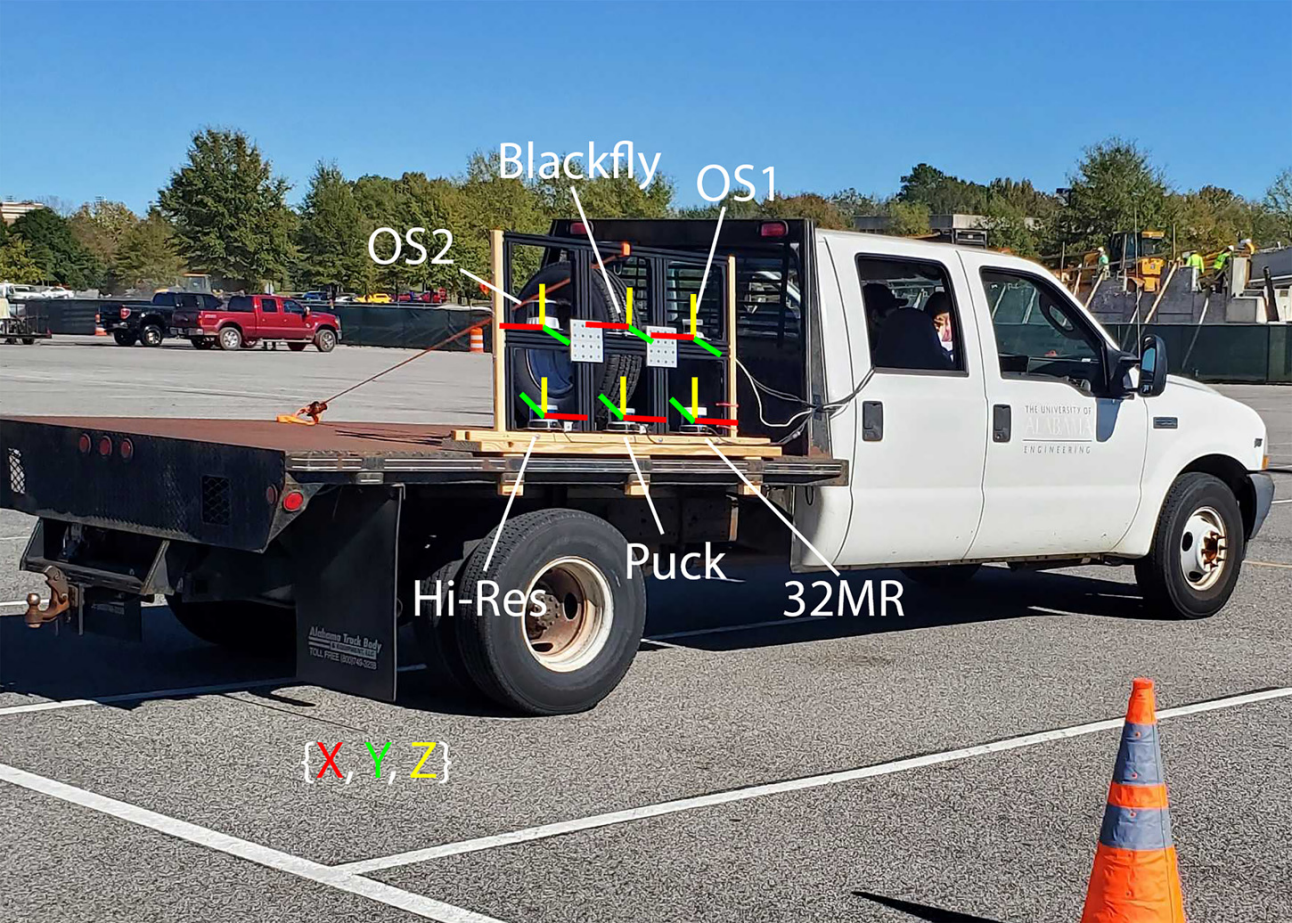
Sensor Arrangement at Sites 3, 4, and 5.

This site is the only one to target trucks. As such, the sensors were pointed at a highway with many other vehicles passing through the FOV, but only trucks have been annotated. For the purposes of labeling, “trucks” were generally defined as any large, industrial vehicle including 18-wheelers, box trucks, dump trucks, etc. However, due to the sparsity of LiDAR data on some truck targets and inconsistencies among labeling personnel, there are a few, random instances of such vehicles not being labeled. Distance to targets of interest ranged from 48m–240m. Sensors were arranged within the sensor mount similar to that of Site 5 (seen in Fig. 7), except mounted to a tripod. The resulting height of the sensors is outlined in Table 4. The position of each sensor relative to the camera’s coordinate frame can be seen in Table 11.

**Table 11:** Sites 3 and 4 relative coordinates.

Sensor	Relative Coordinate (m)
Blackfly	(0, 0, 0)
Puck	(0, -0.015, -0.363)
Hi-Res	(0.381, -0.015, -0.363)
32MR	(-0.381, -0.015, -0.363)
OS1	(-0.381, -0.015, -0.071
OS2	(0.381, -0.015, -0.071)

Due to the volume of scans collected, the data was down sampled for the labeling process. Roughly every fifth scan was labeled. This translates to a labeled scan rate of 1.6 Hz and 2 Hz for the respective sensors, resulting in slightly less synchronized labeled data. This down sampling had no impact on the data rates for collection of raw data. A summary of the total scans collected and those labeled are outlined in Table 12.

**Table 12:** Site 3 data summary.

Sensor	Raw Scans	Labeled Scans
Blackfly	60,633	3,084
Puck	57,735	7,368
Hi-Res	55,290	9,295
32MR	49,235	3,523
OS1	59,061	2,262
OS2	53,797	10,759

### 2.11 LiDAR details

As mentioned in the LiDAR Data section, scans were originally saved as ASCII PCDs. After collection at Site 2, it was found that the larger file sizes of the ASCII format were the primary contributor to the bandwidth issue resulting in the significant data loss discussed in Sites 1 and 2. To solve this issue, the LiDAR driver was modified to save point cloud data as binary PCDs instead. This significantly reduced the file size and bandwidth required, allowing every scan collected to be saved. As such, this enabled data collection at the original rates of 8 Hz and 10 Hz for the Velodyne and Ouster LiDARs respectively.

All sensors had a comparable amount of incomplete scans. The larger amount of incomplete scans outlined in Table 3 is attributed to the hard drive overheating after several hours of continued operation. After allowing the hard drive to cool for several minutes, scan dropout was returned to a nominal amount. After collection, a script was written to convert the PCD files from binary back to ASCII. This methodology was adopted for all future collection sites.

For this site, the static relationship could not be exploited for labeling, as this method would include points belonging to targets that are not of interest. Therefore, every fifth scan was manually labeled.

### 2.12 Camera details

During collection, the camera’s image buffer would occasionally fill up every 4,000–6,000 images. This was due to the large number of images and constant operation of the collection process at a 10 Hz rate. Once the buffer filled, image capturing was momentarily paused to clear the buffer for 15–20 s, and then resumed. Therefore, there are some short gaps in data collection where LiDAR scans have no correlated camera images. To vary the data, the camera was occasionally panned left or right to capture additional viewpoints. Orientation was typically changed for 30–60 min before another change.

When initially labeling the camera data, every image was labeled. This was due to the increased speed of the trucks resulting in enough scene change to warrant labeling. However, after the camera was panned left or right, the view was essentially straight up or down the road (trucks driving towards the camera, instead of across the FOV). When this occurred, the same “pseudo-duplicate” situation arose, and labeling was returned to every 3–7 images based on the annotator’s discretion.

### 2.13 Site 4

This collection occurred near sunset, resulting in scans taken in low light conditions. This new variation in data lets researchers evaluate LiDAR and camera performance at night. Distance to targets of interest ranged from 50 m–160 m. The sensor arrangement within the sensor mount was similar to that of Site 5 (seen in Fig. 7), but again mounted to a tripod. The resulting sensor heights are shown in Table 4. The relative positions are identical to that of the Site 3 collection, seen in Table 11. A summary of the collected and labeled scans is outlined in Table 13.

**Table 13:** Site 4 data summary.

Sensor	Raw Scans	Labeled Scans
Blackfly	2,853	885
Puck	2,544	2,544
Hi-Res	2,459	2,459
32MR	2,458	2,458
OS1	3,075	3,075
OS2	3,070	3,070

### 2.14 LiDAR details

At this site, no changes were made to the driver. Scans were once again saved as a binary PCD and later converted to ASCII. All LiDARs collected data at the base rates indicated in Table 5. Each sensor had a comparable amount of incomplete scans. The low amount of incomplete scans at this site, as outlined in Table 3, indicates that the only scan dropout is now due to inherent sensor inconsistencies. The static relationship was once again exploited to label every scan.

### 2.15 Camera details

The last two trains were captured during sunset and nighttime, respectively. Due to these low light conditions, the exposure, gain, and gamma were adjusted during these trains to improve train visibility. The camera frame rate was the base rate shown in Table 5. Every 3-7 images were labeled, but left to the discretion of the annotator.

### 2.16 Site 5

Site 5 data was collected at the same location as Site 4. This time, the sensor mounting frame was attached to the side of a mobile platform with the resulting sensor heights outlined in Table 4. This can be seen in Fig. 7, along with the coordinate systems for the sensors. For further clarification, the X-axis is aligned with the direction of travel; the Y-axis is perpendicular to the bed of the mobile platform, either pointing towards or away from it; and the Z-axis is always pointed towards the sky. The position of each sensor relative to the camera’s coordinate frame can be seen in Table 14.

**Table 14:** Site 5 relative coordinates.

Sensor	Relative Coordinate (m)
Blackfly	(0, 0, 0)
Puck	(0, -0.015, -363.01)
Hi-Res	(0.381, -0.015, -0.3631)
32MR	(-0.381, -0.015, -0.363)
OS1	(-0.381, -0.015, -0.032)
OS2	(0.381, -0.015, -0.032)

**Fig. 8:**
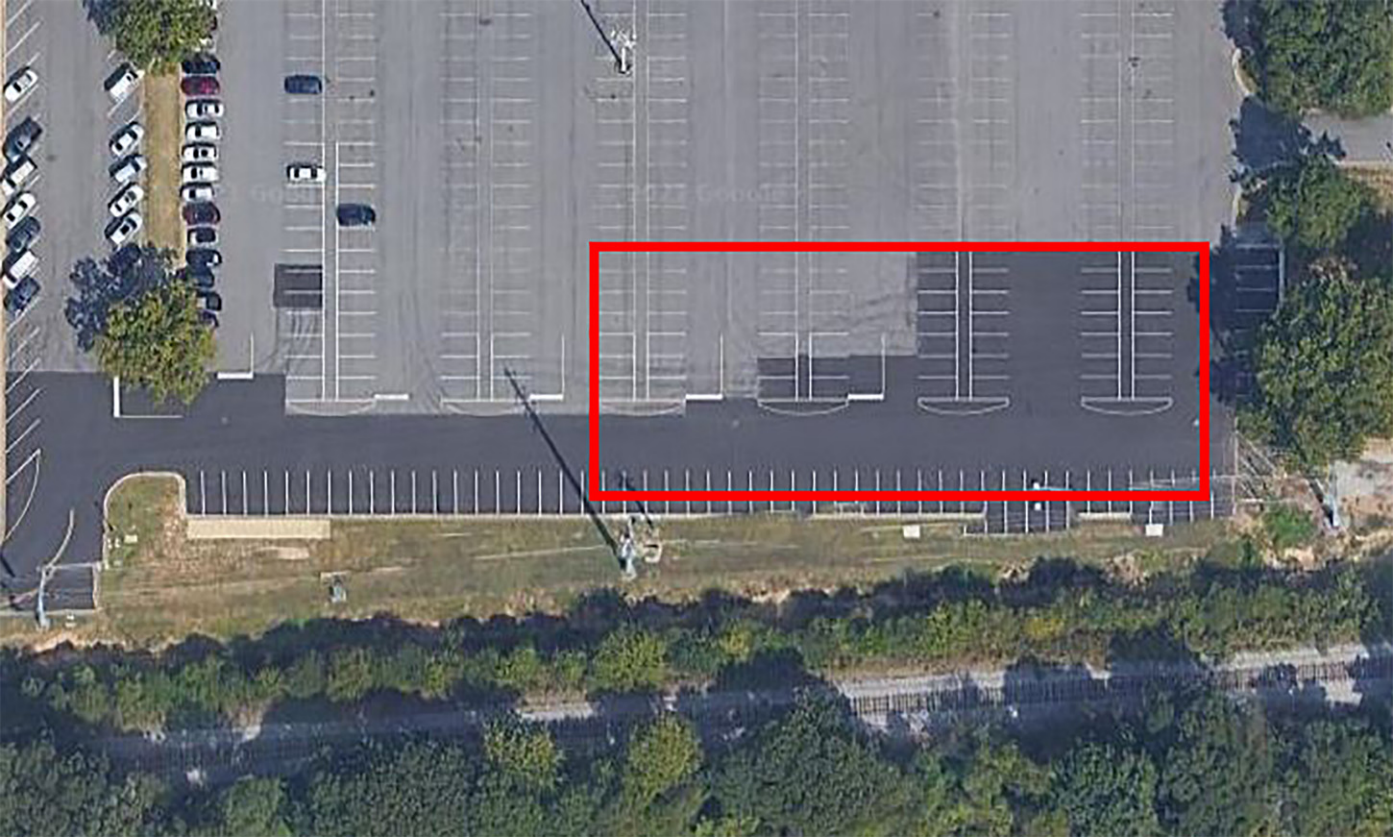
Region of Translation for Site 5.

Once a train approached, collection began and the mobile platform continuously drove around randomly for the duration of the train. This allowed for a large variety of viewpoints to be gathered as the train was passing. Distance to targets of interest ranges from 50 m–160 m. No GPS tracking data is available for exact paths, but the general area can be seen in Fig. 8. Translation speeds ranged from <1 MPH–5 MPH. A summary of the data collected and labeled from Site 5 is outlined in Table 15.

**Table 15:** Site 5 data summary.

Sensor	Raw Scans	Labeled Scans
Blackfly	9,699	1,206
Puck	5,378	1,084
Hi-Res	7,654	1,000
32MR	7,653	1,161
OS1	9,565	1,010
OS2	9,563	997

### 2.17 LiDAR details

Due to the mobile nature of this collection, the LiDARs collected data from their entire 360${}^{\circ }$ FOV. However, small regions are obstructed by the sensor mount itself. During setup for this collection, the Puck sensor was not yet ready when the first train passed. As such, there is no data from this sensor for the first train. It was ready and collecting data for the remaining trains. All LiDAR sensors collected data at their base rates. Each sensor had a comparable amount of incomplete scans. The low amount of incomplete scans can again be attributed to inherent sensor inconsistencies.

Now that the sensors are no longer stationary, there was no longer a static relationship that could be exploited. Roughly 1,000 scans from each sensor were targeted for manual labeling. To create additional variety, 333 scans were taken from the start of one train, middle of another, and end of the last. Train specific scan ranges were randomly selected for each sensor.

### 2.18 Camera details

Due to the nature of the mobile collection, there were times when the camera system could not see the train. This resulted in a higher percentage of negative images than other sites. The camera collected data at its base rate. As with the LiDAR, roughly 1,000 images were targeted from across the start, middle, and end of different trains randomly. The motion of the mobile platform produced enough scenic change between images to avoid the need to skip images.

## CRediT authorship contribution statement

**Maxwell Eastepp:** Methodology, Software, Validation, Investigation, Data curation, Writing – original draft, Writing – review & editing. **Lauren Faris:** Validation, Investigation, Data curation, Writing – original draft, Writing – review & editing. **Kenneth Ricks:** Conceptualization, Methodology, Writing – review & editing, Supervision, Project administration, Funding acquisition.

## Declaration of Competing Interest

This material is based upon work supported by the Army Contracting Command, Contract W909MY-19-C-0020. Any opinions, findings and conclusions or recommendations expressed in this material are those of the authors and do not necessarily reflect the views of the U.S. Army. The authors declare that they have no known competing financial interests or personal relationships which have, or could be perceived to have, influenced the work reported in this article.

## Data Availability

UA_L-DoTT: University of Alabama's Large Dataset of Trains and Trucks – Dataset Repository (Earth/Chem).
